# N-Acetylcysteine Prevents the Spatial Memory Deficits and the Redox-Dependent RyR2 Decrease Displayed by an Alzheimer’s Disease Rat Model

**DOI:** 10.3389/fnagi.2018.00399

**Published:** 2018-12-06

**Authors:** Jamileth More, Nadia Galusso, Pablo Veloso, Luis Montecinos, José Pablo Finkelstein, Gina Sanchez, Ricardo Bull, José Luis Valdés, Cecilia Hidalgo, Andrea Paula-Lima

**Affiliations:** ^1^Faculty of Medicine, Biomedical Neuroscience Institute, Universidad de Chile, Santiago, Chile; ^2^Department of Neurochemistry, Stockholm University, Stockholm, Sweden; ^3^Faculty of Dentistry, Institute for Research in Dental Sciences, Universidad de Chile, Santiago, Chile; ^4^CEMC, Faculty of Medicine, Universidad de Chile, Santiago, Chile; ^5^Pathophysiology Program, Faculty of Medicine, Institute of Biomedical Sciences, Universidad de Chile, Santiago, Chile; ^6^Physiology and Biophysics Program, Faculty of Medicine, Institute of Biomedical Sciences, Universidad de Chile, Santiago, Chile; ^7^Department of Neuroscience, Faculty of Medicine, Universidad de Chile, Santiago, Chile

**Keywords:** amyloid β peptide 1-42, calcium signaling, reactive oxygen species, spatial memory training, antioxidants, early genes, glutathione

## Abstract

We have previously reported that primary hippocampal neurons exposed to synaptotoxic amyloid beta oligomers (AβOs), which are likely causative agents of Alzheimer’s disease (AD), exhibit abnormal Ca^2+^ signals, mitochondrial dysfunction and defective structural plasticity. Additionally, AβOs-exposed neurons exhibit a decrease in the protein content of type-2 ryanodine receptor (RyR2) Ca^2+^ channels, which exert critical roles in hippocampal synaptic plasticity and spatial memory processes. The antioxidant N-acetylcysteine (NAC) prevents these deleterious effects of AβOs *in vitro*. The main contribution of the present work is to show that AβOs injections directly into the hippocampus, by engaging oxidation-mediated reversible pathways significantly decreased RyR2 protein content but increased single RyR2 channel activation by Ca^2+^ and caused considerable spatial memory deficits. AβOs injections into the CA3 hippocampal region impaired rat performance in the Oasis maze spatial memory task, decreased hippocampal glutathione levels and overall content of plasticity-related proteins (c-Fos, Arc, and RyR2) and increased ERK1/2 phosphorylation. In contrast, in hippocampus-derived mitochondria-associated membranes (MAM) AβOs injections increased RyR2 levels. Rats fed with NAC for 3-weeks prior to AβOs injections displayed comparable redox potential, RyR2 and Arc protein contents, similar ERK1/2 phosphorylation and RyR2 single channel activation by Ca^2+^ as saline-injected (control) rats. NAC-fed rats subsequently injected with AβOs displayed the same behavior in the spatial memory task as control rats. Based on the present *in vivo* results, we propose that redox-sensitive neuronal RyR2 channels partake in the mechanism underlying AβOs-induced memory disruption in rodents.

## Introduction

The pathological hallmarks of Alzheimer’s disease (AD) – the most common neurodegenerative disorder and the leading cause of dementia in the elderly – are the presence of amyloid plaques and neurofibrillary tangles, which presumably arise from excessive accumulation and aggregation of amyloid β (Aβ) peptides in the brain of affected individuals. Before the emergence of plaques and/or neurofibrillary tangles, however, independent yet intersecting age-related pathologies have been proposed to cause AD (Tse and Herrup, 2017), including Ca^2+^ dyshomeostasis, excitotoxicity, mitochondrial dysfunction, increased contacts between the ER and the mitochondria, neuro-inflammation and alterations in lipid metabolism (Brito-Moreira et al., 2011; Paula-Lima et al., 2013; Area-Gomez et al., 2018; Olloquequi et al., 2018; Popugaeva et al., 2018). It remains to be established how these early defects relate to the emergence and progression of AD.

The “Amyloid Cascade” theory – currently one of the most accepted hypotheses to explain AD pathogenesis (Selkoe and Hardy, 2016) – considers that accumulation in the brain of Aβ peptide aggregates is the first step leading to dementia. Given their hydrophobic nature, accumulation of Aβ peptides favors their aggregation, giving rise to the emergence of soluble neurotoxic Aβ oligomers (AβOs), insoluble Aβ fibrils and other toxic Aβ aggregates (Reiss et al., 2018). In addition to the fibrils present in senile plaques, and despite their toxicity, current evidence indicates that AβOs play a central role as causative agents of the synaptic dysfunction and memory loss characteristic of AD (Reiss et al., 2018). Moreover, AβOs trigger reactive oxygen/nitrogen species (ROS/RNS) generation (SanMartin et al., 2017; Munoz et al., 2018), decrease neuronal glutathione (GSH) levels, inhibit the activities of antioxidant enzymes and impair mitochondrial function, resulting in neuronal oxidative stress (Turunc Bayrakdar et al., 2014) and non-resolving inflammatory response (Frozza et al., 2018).

Increased ROS generation leads to the oxidative modification of cellular proteins; this process has a profound impact on the pro-inflammatory and the antioxidant responses of brain cells (Rojo et al., 2014). The ryanodine receptor (RyR) channels are endoplasmic reticulum (ER) resident proteins that have been proposed to act as cellular redox sensors (Hidalgo, 2005; Hidalgo and Donoso, 2008). The oxidation/reduction state of highly reactive RyR cysteines determines the Ca^2+^ dependence of RyR single channel activity (Marengo et al., 1998). These findings imply that only RyR channels with oxidized cysteine residues would be responsive to activation by Ca^2+^ – an essential feature of cellular RyR-mediated Ca^2+^-induced Ca^2+^ release (CICR). In neuronal cells, we have shown that RyR channels act as coincidence detectors of the Ca^2+^ increase and ROS production induced by activation of *N*-methyl-D-aspartate (NMDA) receptors (Muñoz et al., 2011; Paula-Lima et al., 2011; Riquelme et al., 2011). By adding a redox component, these findings extend the proposal that RyR channels amplify via CICR the Ca^2+^ signals generated in dendritic spines by activated NMDA receptors (Berridge, 1998; Gleichmann and Mattson, 2011).

Several reports have implicated RyR channels in hippocampal-dependent memory processes. The murine brain expresses the three known mammalian RyR isoforms: type-1 RyR (RyR1), type-2 RyR (RyR2), and type-3 RyR (RyR3) channels (Giannini et al., 1995); of these, RyR2 is the principal RyR isoform expressed in rat hippocampus (Adasme et al., 2011). Training rats in hippocampal-dependent memory tasks increases RyR2 protein content (Zhao et al., 2000; Adasme et al., 2011; Arias-Cavieres et al., 2017; More et al., 2018b), whereas RyR channel inhibition hinders memory retention in mice tested in a radial-arm maze task (Ohnuki and Nomura, 1996). Knockdown of RyR2 or RyR3 – but not of RyR1 – leads to impaired memory retention in a passive avoidance test (Galeotti et al., 2008). Additionally, RyR3 knockout mice exhibit defective spatial learning (Balschun et al., 1999), while selective hippocampal down-regulation of redox-regulated RyR2 channels impairs hippocampal structural plasticity and causes marked defects in the performance of rats in a previously learned spatial memory task (More et al., 2018a).

Previous studies implicated abnormal RyR channel function in AD pathology (Oules et al., 2012; Del Prete et al., 2014), with particular emphasis on RyR3 dysregulation (Stutzmann et al., 2006, 2007; Chakroborty et al., 2009). Of note, downregulation of RyR2 expression in human AD brain postmortem samples occurs very early in the progression of this disease (Kelliher et al., 1999), and abnormalities in RyR2 function have been reported in SH-SY5Y neuroblastoma cells expressing APP harboring the double Swedish mutations (Bussiere et al., 2017) and in rodent transgenic AD models (Lacampagne et al., 2017). Treatment of primary hippocampal neurons with sub-lethal concentrations of AβOs generates low-amplitude but sustained Ca^2+^ signals, which arise from RyR-mediated amplification of Ca^2+^ influx via NMDA receptors (Paula-Lima et al., 2011); these anomalous Ca^2+^ signals lead to mitochondrial and NOX2-mediated ROS generation (SanMartin et al., 2017) and glial activation (Munoz et al., 2018). Also, primary hippocampal neurons exposed to sub-lethal AβOs concentrations exhibit reduced the mRNA and protein levels of RyR2 channels, without affecting the RyR3 levels (Paula-Lima et al., 2011); the antioxidant agents NAC and astaxanthin both reverse the *Ryr2* decrease induced by AβOs *in vitro* (Lobos et al., 2016).

To test *in vivo* if AβOs decrease RyR2 protein content in a redox-dependent manner, we performed bilateral injections of AβOs into the CA3 hippocampal region. We found that AβOs-injected rats displayed spatial memory deficits, exhibited decreased hippocampal protein contents of c-Fos, Arc and RyR2, increased ERK1/2 phosphorylation and increased probability of finding single RyR channels that displayed enhanced activation by Ca^2+^, a characteristic trait of more oxidized RyR channels (Marengo et al., 1998). Additionally, hippocampus-derived mitochondrial-associated membranes (MAM) isolated from these rats exhibited increased contents of RyR2 and other MAM-associated proteins relative to saline-injected rats. Previous NAC feeding preserved hippocampal redox potential; it also prevented the spatial memory deficits and the RyR2 and Arc downregulation induced by subsequent AβOs injections and decreased the frequency of finding RyR channels that respond to Ca^2+^ activation. We conclude that oral ingestion of NAC, by increasing hippocampal GSH content has beneficial effects against the deleterious effects of AβOs on cognition and the redox-dependent down-regulation of RyR2, a protein with a critical role in synaptic plasticity and spatial memory processes (More et al., 2018a).

## Materials and Methods

### Animals

Sprague-Dawley juvenile male rats were procured from the Animal Care Facility of the Faculty of Medicine, Universidad de Chile. Rats were individually housed in a controlled environment with a 12 h light–dark cycle at 21–23°C, with food and water *ad libitum* except when indicated otherwise; all animals were handled daily for 2 weeks before cannulation surgery. The experimental protocols used in this work complied with the “Guidelines for the Care and Use of Mammals in Neuroscience and Behavioral Research,” The National Academies Press, Washington, DC, and were approved by the Bioethics Committee on Animal Research, Faculty of Medicine, Universidad de Chile (protocol #CBA 0755 FMUCH).

### Antibodies

Primary antibodies: mouse monoclonal anti-RyR2 (MA3-916) was from Thermo Fisher Scientific (Waltham, MA, United States); rabbit monoclonal anti-RyR3 (AB9082) and rabbit polyclonal anti-IP_3_ receptor type-1 (IP_3_R1) (AB5882) antibodies were from former Merck-Millipore (Darmstadt, Germany); rabbit polyclonal anti-ACSL4 (SAB2100035) and anti β-actin (A5316) were from Sigma-Aldrich (St. Louis, MI, United States); anti-VDAC (sc-390996), anti-Calnexin (sc-6465) and anti-COX4 (sc-69359) were from Santa Cruz Biotechnology (Dallas, TX, United States); rabbit monoclonal anti-β-amyloid (H31L21) was from Life Technologies (Waltham, MA, United States); rabbit c-Fos polyclonal antibody Ab-5 was from Oncogene (San Diego, CA, United States); Anti-Arc Polyclonal rabbit affinity purified antibody was from Synaptic System (Göttingen, Germany); ERK1/2 and phospho-ERK1/2 antibodies were from Cell Signaling Technologies (Danvers, MA, United States). Secondary antibodies: polyclonal goat anti-mouse IgG-HRP (sc-2005), goat anti-rabbit IgG-HRP (sc-2004) and donkey anti-goat IgG-HRP (sc-2020) were from Santa Cruz Biotechnology (Dallas, TX, United States); polyclonal Alexa 488 goat anti-mouse (A32723) and Alexa Fluor 488 anti-rabbit (A-11034) were from Thermo Fisher Scientific (Waltham, MA, United States); Biotin-SP-AffiniPure goat anti-rabbit IgG (H+L) was from Jackson ImmunoResearch Europe Ltd. (United Kingdom). Molecular weight standards (Precision Plus Protein Standard) were obtained from Bio-Rad (Hercules, CA, United States).

### Preparation of AβOs

The Aβ1-42 peptide was prepared as a dried hexafluoro-2-propanol (HIFP) film and was stored at –80°C for up to 4 months. Before use, peptide films were dissolved in dimethylsulfoxide to yield a 5 mM stock solution, as previously described (Paula-Lima et al., 2011). The 5 mM peptide stock solution was subsequently diluted to 100 μM with cold phosphate buffered saline (PBS) solution (in mM: 137 NaCl, 2.7 KCl, 10 Na_2_HPO_4_, and 1.2 K_2_HPO_4_); after incubation for 24 h at 4°C without stirring, it was centrifuged at 14,000 × *g* for 10 min at 4°C to remove insoluble aggregates (protofibrils and fibrils). The supernatant containing soluble AβOs was transferred to clean tubes maintained at 4°C. Only fresh (up to 2 days-old) preparations of AβOs were used in all experiments.

### NAC Feeding Protocol

Juvenile rats (7-week-old) weighing 150–170 g were fed daily for 21 consecutive days with commercial jelly (1 ml) containing either the antioxidant NAC (200 mg/kg) or vehicle. This oral NAC feeding protocol was maintained during all subsequent procedures, including bilateral cannulation, recovery from surgery, pre-training in the Oasis maze task and testing in this task after intra-hippocampal injections of saline or AβOs.

### Cannulation Surgery

To perform bilateral injections of AβOs or vehicle into the dorsal CA3 hippocampal region, rats were chronically and bilaterally implanted with two 21-gauge stainless-steel cannula guides (Plastics one, Kent, United Kingdom). The coordinates used to target the dorsal CA3, determined according to Paxinos and Watson (2007), were anteroposterior −3.3 from Bregma, lateral ±3.5 mM and 2.7 mM in depth. Cannulas were fixed to the skull with anchors jewelry stainless steel screws and dental acrylic. Antibiotic (Enrofloxacin 19 mg/kg i.p.; Bayer, Shawnee, KA, United States) and anti-inflammatory [Ketophen 0.2 mg/kg i.p.; Drag Pharma (Santiago, Chile)] agents were administered at the end of surgery and during three consecutive days. Rats were left to recover for 7 days after surgery before initiating the pre-training and training sessions, as detailed below.

### Spatial Memory Training and Evaluation

Two different groups of rats, restricted either of food or water to enhance motivation behavior, were exposed to the spatial memory task in the Oasis maze task, a dry-land version of the Morris water maze (Clark et al., 2005). All animals were tested at about 10 weeks of age. Food-restricted rats were placed at 8 week of age (weighing 230–250 g) in the experimental setting; 2 days later, rats underwent surgery to place the cannulas. After a recovery period of 1 week, cannulated rats were subject to food restriction for 7–10 days until they reached 85% of their body weight. At this point, rats (now ∼10-week old) were pre-trained for 3 days in the Oasis maze, followed by a training period of 6 days. Food restriction was maintained in order to keep rats at 85% of their initial weight during all subsequent procedures. Rats of the water-restricted group were habituated at 4 week of age in the experimental settings for 3 weeks, and were fed next with NAC for 3 additional weeks. At the end of the second week of this period, rats underwent surgery. After a recovery period of 1 week, rats (by now 10-week old) were trained in the Oasis maze. Water restriction was initiated 23 h before the start of each pre-training or training session; water was provided *ad libitum* for 1 h after these sessions.

Food or water-restricted rats were pre-trained during three consecutive daily sessions in order to familiarize the animal with the testing environment (circular arena provided with visual cues) and the search for the reward (food or water) in 21 equidistant distributed wells. In the pre-training sessions, performed at Zeitgeber time (ZT) 7, all wells contained the reward. After the completion of the third pre-training session, animals received (at ZT 9) the first out of three sequential 0.5 μL bilateral injections of AβOs (≈20 pmol) or saline. Injections were completed in a period of 48 h, as detailed in the complete scheme of injections and training sessions illustrated in Supplementary Figure 1. The ensuing training tasks, which started 24 h after the first AβOs injection, entailed searching for the reward in one out of 21 wells during six daily sessions. Each session encompassed 15 trials of 1 min duration each, performed at 20–30 s inter-trial intervals. The reward was placed in a different well in each session but was kept in the same position during the 15 trials. Animal behavior was recorded with a video camera in the zenithal position. The position of the animal was monitored continuously during the tests, and the navigation trajectory was reconstructed and analyzed with a customized MATLAB (MathWorks) routine. One hour after the end of the sixth session, animals underwent euthanasia by decapitation, and the hippocampus was collected for immunoblot analysis. Alternatively, animals were perfused for immunofluorescence or immunohistochemistry assays of plasticity-related proteins, as detailed below.

### Immunofluorescence Assays

Adult rats were transcardially perfused with 300 ml of saline flush and 300 ml of 4% paraformaldehyde in 0.1 M PBS, pH7.4 (Sigma, St. Louis, MI, United States) 1 h after the last test session. The brain was removed, postfixed in 4% paraformaldehyde for 2 h at room temperature, and subsequently incubated for 72 h at 4°C in a solution containing 30% sucrose, 0.002% sodium azide for cryopreservation. Brains were cut in the coronal plane with a sliding frozen microtome (−30°C) at 40 μM thickness. Free-floating sections were immersed for 2 h at room temperature in PBS containing 0.25% Triton X-100 (PBS-TX) plus 3% donkey serum and were incubated overnight at 4°C with PBS-TX containing primary antibodies (anti-RyR2 1:50, anti-RyR3 1:100, or anti-Aβ 1:50). Sections were washed 4-times for 5 min in PBS and were incubated next for 2 h with secondary fluorescent antibodies (Alexa 488 1:300 or Alexa Fluor 488 1:300).

Brain tissue slices were washed in PBS, mounted on glass slides and covered with mounting medium. The Hoechst reagent (1:10,000, Sigma, St. Louis, MI, United States) was employed for nuclear staining. A z-stack of 1.5 μM sections was captured from different hippocampal regions (CA1, CA3, and DG), in a confocal microscope (Nikon C2+, Melville, NY, United States). Fluorescence intensity was determined with the confocal microscope NIS-Elements software viewer 4.0, and with ImageJ free viewer software.

### Immunohistochemistry

Free-floating hippocampal sections were incubated for 30 min in PBS containing 0.3% H_2_O_2_, washed 2 times for 10 min in PBS and incubated 1 h at room temperature in blocking solution (0.4% Triton X-100, 0.02% sodium azide, and 3% normal goat serum in PBS). Sections were incubated overnight (16–18 h) at room temperature with blocking solution containing primary c-Fos antibody (1:20,000). Sections were washed 6 times for 10 min with PBS and were incubated with the secondary antibody (Biotin-SP-conjugated Affinipure goat anti-rabbit IgG (H + L); 1:1000). After washing 4 times for 10 min with 0.01 M PBS, sections were incubated for 1 h in Vectastain ABC Elite Kit (Vector Laboratories, Burlingame, CA, United States; 1:500). Finally, sections were rinsed 3 times for 10 min with 0.01 M PBS and were revealed with 0.05% 3–3′-diaminobenzidine hydrochloride (DAB) and nickel chloride, producing an enhanced dark blue reaction product for c-Fos. Immunostaining for Arc was performed similarly, using primary antibodies against Arc (1:5,000) and DAB staining without nickel chloride intensification, which yielded a brown cytoplasmic precipitate corresponding to Arc.

### MAM Isolation

The whole hippocampi were extracted from AβOs-injected or saline-injected rats at ∼10 weeks of age (*n* = 6 in each case), pooled, weighed and diluted to 4% w/v in solution A (in mM: 320 sucrose, 300 KCl, 1 NaHCO_3_, plus protease inhibitors). After homogenization with a glass/Teflon homogenizer, the resulting suspension was centrifuged at 1,400 × *g* for 10 min at 4°C, and the supernatant was collected and reserved on ice. The pellet, re-suspended in four volumes of solution A, was centrifuged at 710 × *g* for 10 min at 4°C. The two supernatants were pooled and kept on ice. The pellet containing nuclei and non-lysed cells was re-suspended as above and centrifuged at 13,800 × *g* for 10 min at 4°C. This procedure was repeated three times. The resulting pellet contained an enriched mitochondrial fraction. All collected supernatants, corresponding to the cytosolic and endoplasmic reticulum fractions, were pooled together and centrifuged at 135,000 × *g* for 30 min at 4°C in a Beckman preparative ultracentrifuge (model Optima XPN 90K, 50.2 Ti Rotor). The resulting pellet corresponded to the endoplasmic reticulum fraction, while the supernatant corresponded to the cytosolic fraction. The pellet containing an enriched mitochondrial fraction was re-suspended in 1 ml of solution B (in mM: 320 sucrose, 1 NaHCO_3_), loaded on top of a discontinuous sucrose gradient formed by equal volumes of 29, 34, and 41% sucrose (w/v) in 1 mM NaHCO_3_, and centrifuged at 13,500 × *g* for 2 h at 4°C. Fractions were collected, and the pellet containing mitochondria was re-suspended in 2 ml of solution A, containing protease inhibitors. For isolation of the MAM fraction, the re-suspended mitochondria-containing pellet was poured on top of 30% Percoll and centrifuged at 135,000 × *g* for 30 min at 4°C in a Beckman preparative ultracentrifuge (model Optima XPN 90K 50.2 Ti Rotor). After centrifugation, two different bands were obtained, the lower band corresponded to the mitochondrial fraction and the upper band contained the MAM fraction. Fractions were collected separately, diluted in 4 ml of solution A and centrifuged at 6,300 × *g* for 10 min at 4°C. The pellets from the tubes containing the respective MAM fractions were discarded, and the supernatants were added to 0.3 ml of isolation medium (in mM: 250 Mannitol, 0.5 EGTA, 5 HEPES, pH 7.4, 0.1% BSA) and centrifuged at 135,000 *g* for 1 h at 4°C. The resulting pellets, containing the MAM fractions, were re-suspended in 0.2 ml isolation medium and stored at −80°C prior to immunoblot analysis. Protein concentration was determined by the turbidimetric sulfosalicylic acid method (Henry et al., 1956).

### Western Blot Analysis

Western blot analysis of hippocampal tissue homogenates was performed as previously described (Arias-Cavieres et al., 2017). The isolated rat hippocampus was homogenized with a glass/Teflon homogenizer at 4°C in 200 μL of lysis buffer (in mM: 300 sucrose, 2 EDTA, 2 EGTA, 1 BAPTA, 20 MOPS-Tris pH 7.5 plus 1% NP40, 0.1% SDS) containing protease inhibitors (Calbiochem, La Jolla, CA, United States). The homogenate was maintained at 4°C, sonicated 4 times for 20 s at 20 s intervals, and incubated on ice for 10 min. Protein concentration was determined by the turbidimetric sulfosalicylic acid method (Henry et al., 1956). Samples were resolved by electrophoresis in 3–8% Tris-Acetate gels (Criterion^TM^ XT Precast Gel, Bio-Rad, Hercules, CA, United States); gels were immersed in Tris-Tricine buffer and run for 3 h at 80 V. The protein bands were transferred to PDVF membranes (Millipore Corp., Bedford, MA, United States) using the Transfer-Blot R Turbo System (Bio-Rad, Hercules, CA, United States), following the supplier’s instructions. PVDF membranes were incubated for 1 h at room temperature using as blocking solution Tris-buffered Saline (TBS) with 5% fat-free milk for RyR2 detection, or 5% bovine serum albumin (BSA) for RyR3 detection. The membranes were incubated overnight at 4°C in blocking buffers with specific primary antibodies (anti-RyR2 1:1,000, anti-RyR3 1:2,000); anti-β-actin (1:12,000) was used as loading control. Image acquisition and densitometric analysis of band density were performed with the Image Lab software, Chemidoc^TM^ MP System (Bio-Rad, Hercules, CA, United States).

### Glutathione Determination

The levels of GSH and oxidized glutathione (GSSG) in whole hippocampus homogenates were determined as previously described (Griffith, 1980). In brief, the hippocampus was isolated from four groups of rats: control, AβOs, NAC/AβOs, NAC/saline, after exposure to the spatial memory task. The hippocampus was homogenized and deproteinized with sulfosalicylic acid. To determine the total concentration of glutathione (GSH + GSSG), samples were treated with glutathione reductase to convert GSSG into GSH, and diluted in reaction solution (6 mM DTNB, 0.3 mM NADPH, 1 mM EDTA, 50 mM phosphate buffer, pH 7.5); the resulting absorbance was detected at 410 nm. To determine the GSSG levels of the above protein-free solution, reduced GSH was derivatized with 2-vinylpyridine and neutralized with trimethylamine; the resulting solution was treated with glutathione reductase to convert GSSG into GSH, which was determined as above (Griffith, 1980). The GSH levels were calculated as the difference between the total concentrations of glutathione and GSSG.

### Determination of RyR Single Channel Activity

Juvenile rats (9-week old) were injected bilaterally in the CA3 region with 1 μL AβOs or saline and the whole hippocampus was isolated 48 h later. The hippocampus was homogenized in sucrose buffer with protease inhibitors (0.3 mM sucrose, 20 mM MOPS-Tris, pH 7.0, 0.4 mM benzamidine, 10 μg/ml trypsin inhibitor; 10 μg/ml pepsatin) and centrifuged at 5,000 × *g* during 20 min. The supernatants were centrifuged at 100,000 × *g* for 1 h; the resulting pellets were resuspended in sucrose buffer with protease inhibitors and frozen in aliquots at −80°C.

Channel recordings were obtained as reported previously (Marengo et al., 1996, 1998; Bull et al., 2003, 2008). During channel recording at 22 ± 2°C, the *cis*-(cytoplasmic) solution contained 0.5 mM Ca^2+^-HEPES, and 225 mM HEPES–Tris, pH 7.4, while the *trans* (intrareticular) solution contained 40 mM Ca^2+^–HEPES, 15 mM Tris–HEPES, pH 7.4. The lipid bilayer was held at 0 mV. Current data were filtered at 400 Hz (−3 dB) using an eight-pole low-pass Bessel type filter (902 LPF; Frequency Devices) and digitized at 2 kHz with a 12-bit analog-to-digital converter (LabMaster DMA Interface; Scientific Solutions) using the AxoTape (Molecular Devices) commercial software. Fractional open time (*P*_o_) values were computed using the pCLAMP (Molecular Devices) commercial software. According to their extent of activation by [Ca^2+^], channels were classified as low (*P*_o_ < 0.1 at all [Ca^2+^] tested), moderate (*P*_o_ > 0.1 in the [Ca^2+^] range of 10–100 μM, with reduction in *P*_o_ at 500 μM [Ca^2+^]), or high (*P*_o_ near 1.0 between 3 and 500 μM [Ca^2+^]) activity channels (Marengo et al., 1996; Bull et al., 2003, 2008). Because low and moderate activity channels gated with fast kinetics between closed and open states, *P*_o_ values were calculated as the ratio between the mean current of the entire channel recording and the single-channel current amplitude measured in long-lasting (>30 ms) fully open events (Bull et al., 2003).

### Statistical Analysis

Results are expressed as mean ± SE. Statistical analysis between groups was performed with Two-way ANOVA, One-way ANOVA followed by Holm-Sidak *post hoc* test, as indicated in the respective figure legends. Comparison between two groups was performed by two-tailed Student’s *t*-test. All statistical analyses were performed using SigmaPlot version 12.0.

## Results

### Intra-Hippocampal AβOs Injections Impair Spatial Memory

To test if bilateral intra-hippocampal AβOs injections disrupt spatial learning and memory processes, we injected three times the animals in the CA3 region with AβOs in a period of 48 h, starting 24 h before training (see Supplementary Figure 1A). Rats were tested in six consecutive daily sessions in the Oasis maze task. During the daily training sessions composed of 15 trials, rats injected with AβOs exhibited substantial deficits in task performance in comparison to control animals.

**FIGURE 1:**
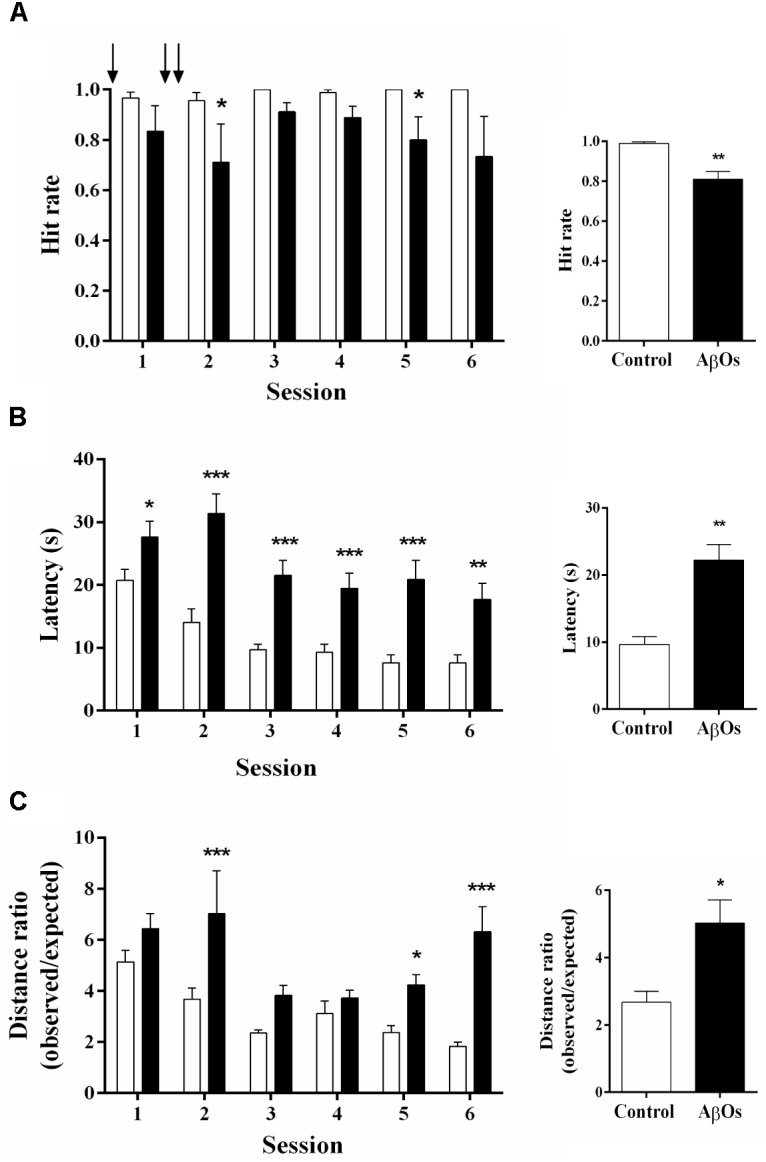
Intra-hippocampal AβOs injections impair spatial memory. The spatial memory of food restricted rats injected intra-hippocampus with AβOs or saline was assessed in the Oasis maze task. **(A)** Hit rate, which corresponds to the number of times the rat found the reward (food) in the 15 trial-session, performed by animals injected with saline (white bars) or AβOs (black bars). The injections are indicated by arrows. **(B)** Latency, which corresponds to the time (in seconds) taken by the animals to find the reward. **(C)** Quantification of the ratios between observed/expected distances performed by animals injected with saline (control) or AβOs. Values represent mean ± SE (*n* = 6 for each group). Results were statistically analyzed by Two-way ANOVA, followed by Holm-Sidak *post hoc* test; ^∗^*p* < 0.05; ^∗∗^*p* < 0.01; and ^∗∗∗^*p* < 0.001.

After finishing the injections (sessions 2 to 6), AβOs-injected rats displayed lower hit rates (Figure 1A, left panel), defined as the number of times the rat found the reward over the 15 trials, with significantly lower average values compared to the controls (Figure 1A, right panel). The decreased hit rate exhibited by rats injected with AβOs contrasted with the behavior of saline-injected rats, which displayed nearly 100% success in this task. Likewise, the average latency to find the reward in sessions 2 to 6 was significantly lower in the control group (Figure 1B, left panel) compared to the group injected with AβOs (Figure 1B, right panel). The ratio of the observed over the expected (shortest) distance covered by the animals while searching for the reward was higher in the AβOs-injected group (Figure 1C, left panel) with respect to the control group (Figure 1C, right panel).

After performing the last session in the Oasis maze, rats were euthanized, and their brains were isolated and analyzed. Nissl staining of hippocampal slices showed that the cannula was placed right over the CA3 region (Supplementary Figure 1B). Confocal microscopy of slices immunostained with anti-Aβ antibody showed the presence of Aβ in the hippocampus of AβOs-injected rats (Supplementary Figure 1C).

These combined results show that our protocol of AβOs injections into the CA3 hippocampal region produced significant impairments in rat performance of a hippocampus-dependent spatial memory task.

### Intra-Hippocampal AβOs Injections Down-Regulate c-Fos, Arc and RyR2 Hippocampal Protein Contents

**FIGURE 2:**
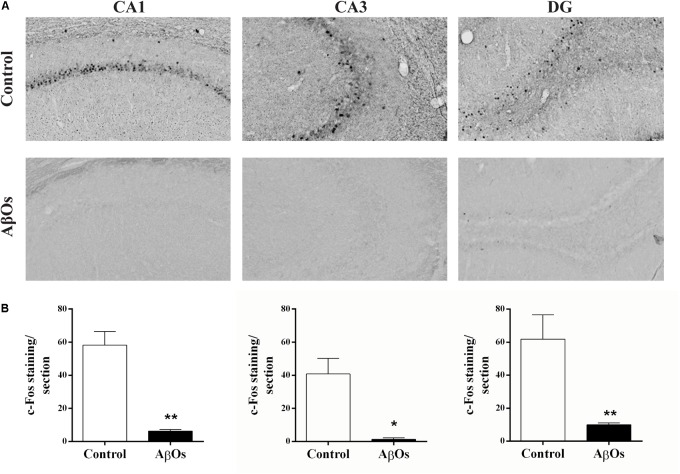
Hippocampal c-Fos expression in AβOs- or saline-injected rats trained in Oasis maze task. **(A)** Representative optical microscopy images of CA1, CA3, and DG hippocampal sections from AβOs- or saline-injected food restricted rats; sections fixed 1 h after the completion of the last training session were stained for c-Fos (see Materials and Methods). **(B)** Quantification of total c-Fos staining in coronal sections from six animals. Values are expressed as mean ± SE (*n* = 6). Statistical analysis was determined by two-tailed unpaired Student’s *t*-test; ^∗^*p* < 0.05 and ^∗∗^*p* < 0.01.

Spatial learning induces the expression of the immediate-early genes c-Fos and Arc in rat hippocampus (Vann et al., 2000; Guzowski et al., 2001; Ramirez-Amaya et al., 2005; Mamiya et al., 2009). Conversely, the hippocampus and cerebral cortex from an experimental rodent model of the familial AD, the APPswe/PS1ΔE9 transgenic mice that display mutations in APP and presenilins, exhibit decreased basal and novelty-induced mRNA levels of *c-Fos* and *Arc* (Christensen et al., 2013). We tested next whether intra-hippocampal AβOs injections modified c-Fos and Arc protein contents in the hippocampus of rats 1 h after the last spatial memory training session (see Supplementary Figure 1A). In coronal sections from saline-injected rats, we found the typical c-Fos localization in the nuclei of some cells from the CA1, CA3, and DG regions, as shown in the representative images illustrated in Figure 2A.

Rats injected with AβOs exhibited a marked decrease in c-Fos protein immunoreactivity in all three hippocampal regions (Figure 2A). Quantification of c-Fos staining confirmed the c-Fos decrease in all three hippocampal regions from AβOs-injected rats, which showed significant differences relative to their respective controls (Figure 2B). In addition, the representative images of hippocampal sections from AβOs-injected rats illustrated in Supplementary Figure 2A displayed a striking reduction in Arc immunoreactivity in the DG region relative to the control. In contrast, the CA1 and CA3 regions from saline-injected or AβOs-injected rats did not display detectable Arc immunoreactivity after exposure to the spatial memory task, in the Oasis maze. The quantification of DG images, shown in Supplementary Figure 2B, yielded average values (*n* = 6) significantly lower for the AβOs-injected compared to the saline-injected controls, respectively. These results indicate that AβOs-injected rats presented a marked reduction in the protein content of c-Fos and Arc, two early gene products that, as mentioned above, have a crucial role in hippocampal-dependent synaptic plasticity and memory.

**FIGURE 3:**
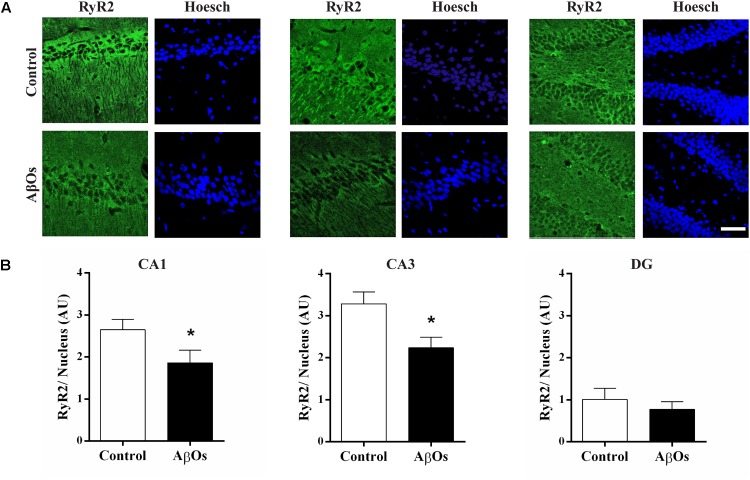
AβOs injections decrease RyR2 immunolabeling. **(A)** Representative confocal microscopy 3-D projections (20 slices, 1.5 μM each) of CA1, CA3, and DG hippocampal regions isolated from food restricted rats exposed to the memory training protocol, fixed after the sixth training session and labeled with a RyR2 antibody (green) and DAPI-nuclear staining (blue). Scale bar: 20 μM. **(B)** Quantification of total RyR2 fluorescence normalized by total DAPI nuclear staining. Values are expressed as mean ± SE (*n* = 6). Statistical analysis was determined by two-tailed unpaired Student’s *t*-test; ^∗^*p* < 0.05.

Hippocampal coronal sections immunostained for the RyR2 protein (More et al., 2018a) exhibited lower RyR2 immunostaining in the CA1 and CA3 hippocampal regions from AβOs-injected relative to saline-injected trained rats, as shown in the representative images illustrated in Figure 3A. Quantification of images revealed that RyR2 protein content was significantly decreased in the CA1 and CA3 hippocampal regions from rats injected with AβOs compared to controls; no significant changes were observed in the DG region (Figure 3B).

**FIGURE 4:**
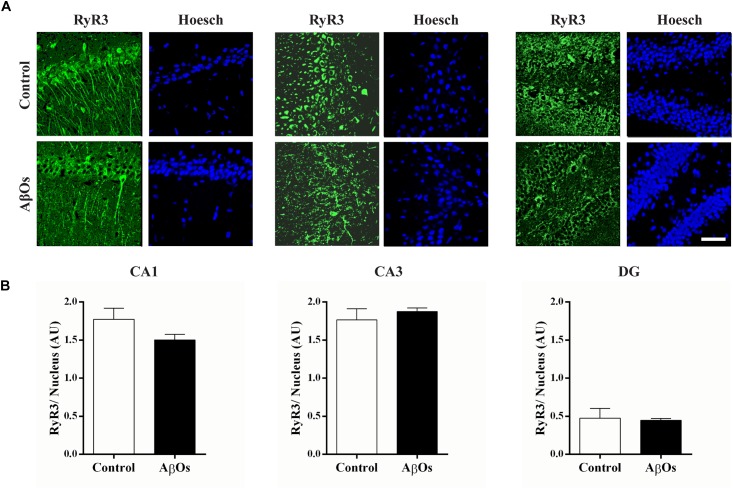
AβOs-injections do not modify RyR3 immunolabeling. **(A)** Representative confocal microscopy 3-D projections (20 slices, 1.5 μM each) of CA1, CA3, and DG hippocampal regions from food restricted trained rats, fixed after the sixth training session and labeled with a RyR3 antibody (green) and DAPI staining for the nucleus (blue). Scale bar 20 μM. **(B)** Quantification of total fluorescence of RyR3 normalized by the total fluorescence of the DAPI nuclear staining. Statistical analysis was determined by two-tailed unpaired Student’s *t*-test.

Immunostaining with a RyR3 specific antibody did not reveal differences in RyR3 protein content between the two groups, as observed in the representative immunofluorescence images (Figure 4A) and the quantification of the images from six independent experiments (Figure 4B).

### Intra-Hippocampal AβOs Injections Increase the Content of RyR2 and Other MAM-Associated Proteins

**FIGURE 5:**
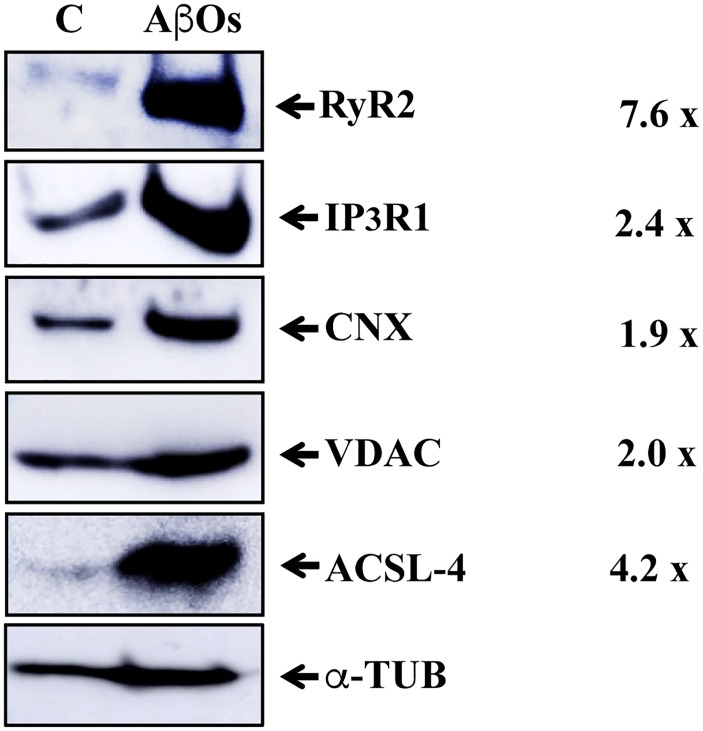
Hippocampal-derived MAM fractions contain RyR2, which is enriched in the MAM fraction from AβOs-injected rats. Western blot images of MAM fractions isolated from rat hippocampus (see Materials and Methods). The ER proteins analyzed were Calnexin (CNX), IP_3_R1, and RyR2. The inner and outer mitochondrial membrane proteins analyzed were COX1 and VDAC1, respectively; the characteristic MAM protein was ACSL-4. C, control, saline-injected; AβOs, AβOs-injected. The column at right indicates the ratios between band densities of each MAM protein (AβOs/control) normalized to b-tubulin protein levels.

We reported recently that primary hippocampal cultures exposed to AβOs exhibit significant mitochondrial Ca^2+^ overload as a result of enhanced RyR2-mediated Ca^2+^ release (SanMartin et al., 2017). Accordingly, we examined whether hippocampal MAM fractions from rats injected with saline or AβOs contained the RyR2 isoform. Western blot analysis of MAM fractions isolated from the pooled hippocampus tissue from six animals (Figure 5) shows that the MAM fraction from rats injected with saline contains known MAM-associated proteins, including the ER-resident proteins IP_3_R1 and calnexin (CNX) and the mitochondrial VDAC1 proteins. This MAM fraction also presented weak immunoreactivity for RyR2 and acyl-CoA synthetase long-chain family member 4 (ACSL-4), a known MAM marker (Wieckowski et al., 2009). Remarkably, the MAM fraction from rats injected with AβOs exhibited a substantial increase in RyR2 protein content, in addition to the higher protein levels of IP_3_R1 and VDAC. These results confirm the increased in IP_3_R and VDAC previously reported in human AD brain and related AD transgenic mouse and neuronal cell models (Hedskog et al., 2013). A slight and a huge increase in CNX and ACSL-4 protein content, respectively, were found in the MAM fraction from rats injected with AβOs (Figure 5). We interpret these combined novel findings as an indication that the RyR2 isoform is a component of the hippocampal MAM fraction, and that AβOs injection increases MAM protein content and causes a substantial increment in MAM-associated proteins, including RyR2, IP_3_R1, CNX, and ACSL-4.

### Intra-Hippocampal AβOs Injections Modified the Ca^2+^ Activation Profile Displayed by Single RyR Channels

**FIGURE 6:**
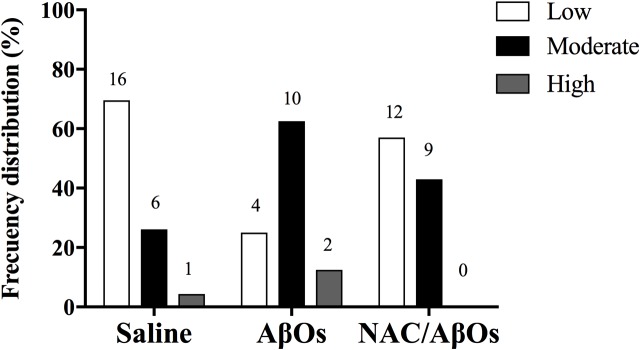
AβOs increase the activity of single RyR channels in planar bilayers. The graph indicates the frequency distribution of incorporation of single channels with low (white bars), moderate (black bars), or high (gray bars) Ca^2+^-induced activity. Channels were incorporated into bilayers from vesicle preparations of rat hippocampus isolated 48 h after AβOs or saline (control) stereotaxic injections into the CA3 hippocampal region performed in control or NAC-fed rats. Numbers on top of each bar indicate the number of single channels included in each condition; *n* = 3 animals for each experimental group.

As previously reported in rat brain cortex homogenates (Bull et al., 2008), single RyR channels from control or AβOs injected hippocampi displayed three different responses to increasing cytoplasmic [Ca^2+^], which we classify as low, moderate, or high activity channels. These different channel responses reflect RyR channel redox state. Highly reduced channels are barely activated by Ca^2+^; increasing RyR oxidation generates the moderate activity response whereas further RyR oxidation elicits the high activity response (Marengo et al., 1998). These three types of response to cytoplasmic [Ca^2+^] changes were indistinguishable for single RyR channels from AβOs injected or control hippocampi, but displayed different frequency distributions (Figure 6). RyR channels from control hippocampi displayed most frequently the low activity response (70%), followed by the moderate (26%) and high (4%) activity responses to varying [Ca^2+^]. In contrast, channels from AβOs injected hippocampi displayed a different frequency distribution, and exhibited most frequently the moderate activity (63%) response to [Ca^2+^], followed by the low (25%) and high (12%) activity responses. Therefore, AβOs injections induced a marked change in the emergence of the different RyR channel responses to [Ca^2+^]: it reduced the fraction of low activity channels from 70 to 25%, increased the fraction of moderate activity channels from 26 to 63% and of high activity channels from 4 to 12%. These results show, for the first time, that AβOs injections intra-hippocampus increased approximately two to threefold the probabilities of finding more oxidized RyR channels that displayed moderate and high activity, respectively, in response to cytoplasmic Ca^2+^ when compared with controls, which seldom yielded high activity channels. The implications of these findings for RyR channel redox state are presented in the Discussion section.

### NAC Consumption Prevents the Cognitive Impairments Induced by Intra-Hippocampal AβOs Injections

The antioxidant agent NAC is a physiological precursor of the synthesis of cellular GSH that in primary hippocampal neurons prevents the RyR-mediated Ca^2+^-induced Ca^2+^ release (Paula-Lima et al., 2011; Sanmartin et al., 2012), as well as the RyR2-dependent increase in mitochondrial Ca^2+^ uptake and the enhanced superoxide anion and H_2_O_2_ production induced by AβOs (SanMartin et al., 2017). Therefore, we tested whether NAC consumption improved the cognitive impairment displayed by AβOs-injected rats while performing the Oasis maze task. To this aim, we worked with 4 experimental groups. Two groups were fed with NAC; one was injected with AβOs (NAC/AβOs) and the other with saline (NAC/Saline). The other two groups, not fed with NAC, were injected either with AβOs or saline. In order to ensure adequate NAC feeding, we restricted the animals of water instead of food (see Materials and Methods).

**FIGURE 7:**
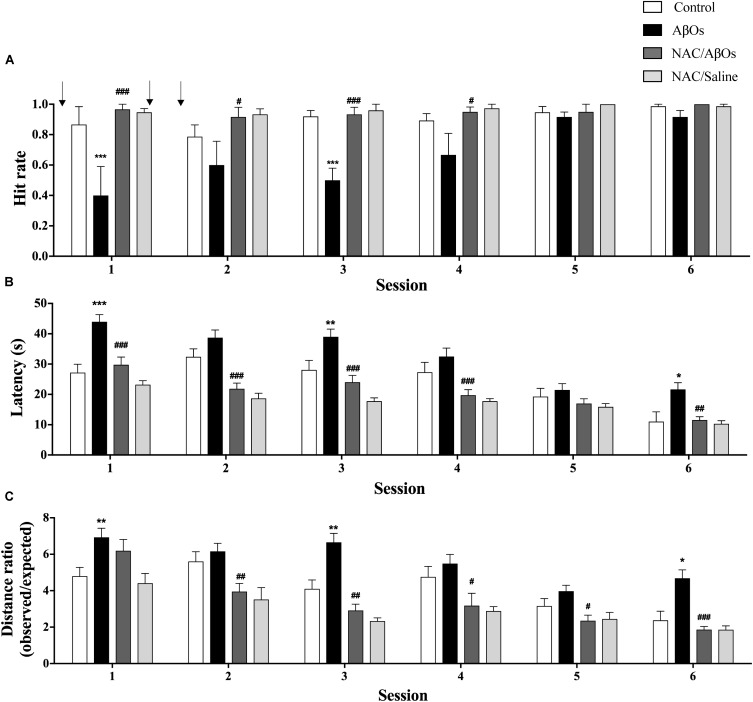
Oral NAC supplementation prevents the spatial memory impairment caused by intra-hippocampal AβOs injections. Spatial memory of water deprived rats fed with NAC or vehicle for 21 days and injected intra-hippocampus with AβOs or saline was assessed in the Oasis maze task. **(A)** Quantification of hit rates for four groups of animals: injected with saline (white bars), with AβOs (black bars), NAC-fed and injected with AβOs (dark gray bars), and NAC-fed and injected with saline (light gray bars). The injections are indicated by arrows. **(B)** Quantification of the latency in each daily session for the four groups defined in **A**. **(C)** Distance ratios for the four experimental groups in each daily session. Values represent mean ± SE (*n* = 6). Statistical analysis was performed by two-way ANOVA, followed by Holm-Sidak *post hoc* test; ^∗^*p* < 0.05, ^∗∗^*p* < 0.01, and ^∗∗∗^*p* < 0.001 compared to control group; ^#^*p* < 0.05, ^##^*p* < 0.01, and ^###^*p* < 0.001 compared to the AβOs-injected group.

The group injected with AβOs displayed a significant decrease in the hit rates in sessions 1 to 4 (Figure 7A) that were significantly lower than those displayed by the saline-injected control group in all six sessions. Nonetheless, AβOs-injected rats displayed similar hit rates as the other three groups in sessions 5 and 6, an indication that they eventually managed to learn the task. In all six daily sessions rats from the NAC/AβOs group displayed average values not significantly different from those exhibited by rats from the NAC/Saline group and by saline-injected rats (Figure 7A), a sign that animals of the NAC/AβOs group, despite having been injected with AβOs successfully learnt the task as well as the other two groups. Albeit from session 1 to session 6 all four groups displayed a decrease in the latency times to find the reward, in all six sessions the latency times were higher for the AβOs group relative to the other three groups (Figure 7B). These results confirm the impaired memory capacity of rats from the AβOs-injected group and show that rats from the NAC/AβOs group presented similar decreases in average latencies from session 1 to 6 as those exhibited by rats from the saline-injected or the NAC/Saline groups (Figure 7B). Likewise, in all six sessions, the ratios between the observed/expected distances were higher for the AβOs-injected group relative to the other three groups (Figure 7C), albeit all groups displayed a decrease in this parameter as a function of increasing session number. Of note, the decrease in distance ratios for the NAC/AβOs and the NAC/Saline groups was similar and somewhat steeper than the decrease displayed by the saline-injected control group (Figure 7C).

In addition, while searching for the reward rats injected with AβOs performed a longer, and therefore less efficient navigation path compared to the expected shortest path (straight line) than saline-injected rats. In contrast, the path taken by the NAC/AβOs group was more efficient and similar to the navigation trajectory performed by the control and the NAC/Saline groups (Supplementary Figure 3). These results show that NAC feeding prevented the deleterious effects of AβOs injection on rat navigation in the Oasis maze spatial memory task in all three behavioral parameters evaluated and even improved to some extent the performance of NAC-fed rats relative to saline-injected rats.

### NAC Consumption Increased Total Cellular Glutathione and GSH/GSSG Ratios and Partially Restored the Ca^2+^ Activation Profile of Single RyR Channels

**FIGURE 8:**
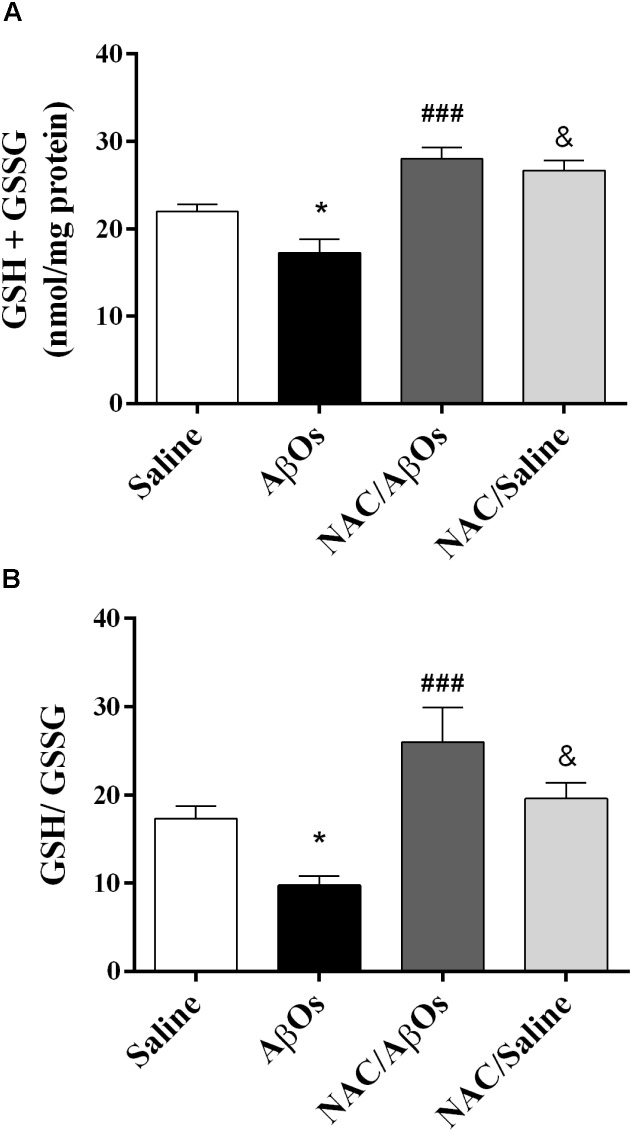
NAC supplementation overrides the hippocampal GSH decrease induced by intra-hippocampal AβOs injections. **(A)** Biochemical determination of total glutathione (GSH+GSSG) content in the hippocampus from four groups of water-deprived trained rats: injected with saline (Saline), injected with AβOs (AβOs), NAC-fed and injected with AβOs (NAC/AβOs), and NAC-fed and injected with saline (NAC/Saline). **(B)** Determination of the intracellular GSH/GSSG ratio in rat hippocampus. Values are expressed as mean ± SE (*n* = 4). Statistical analysis was determined by one-way ANOVA followed by Holm-Sidak *post hoc* test; ^∗^*p* < 0.05 respect to control; ^###^*p* < 0.001 respect to AβOs; ^&^*p* < 0.05 respect to NAC/AβOs.

As illustrated in Figure 8, AβOs-injected rats displayed after exposure to the task lower total glutathione levels and lower GSH/GSSG ratios relative to saline-injected rats, while rats from the NAC/AβOs and the NAC/saline groups displayed after learning higher total glutathione levels and higher GSH/GSSG ratios relative to AβOs-injected rats. These findings show that NAC feeding preserves the endogenous antioxidant levels in the hippocampus of trained rats injected with AβOs or saline.

In addition, single RyR channels isolated from the hippocampus of AβOs-injected NAC-fed rats displayed with slightly higher frequency (57%) the low activity response to [Ca^2+^], followed by the moderate activity (43%) response. Of note, out of 21 channels analyzed, none of them displayed the high activity response (Figure 6). This frequency distribution is intermediate between the frequency distributions displayed by single RyR channels from saline-injected and AβOs-injected rats (Figure 6).

### NAC Feeding Prevents AβOs-Induced Arc and RyR2 Down-Regulation and AβOs-Induced ERK1/2 Phosphorylation

**FIGURE 9:**
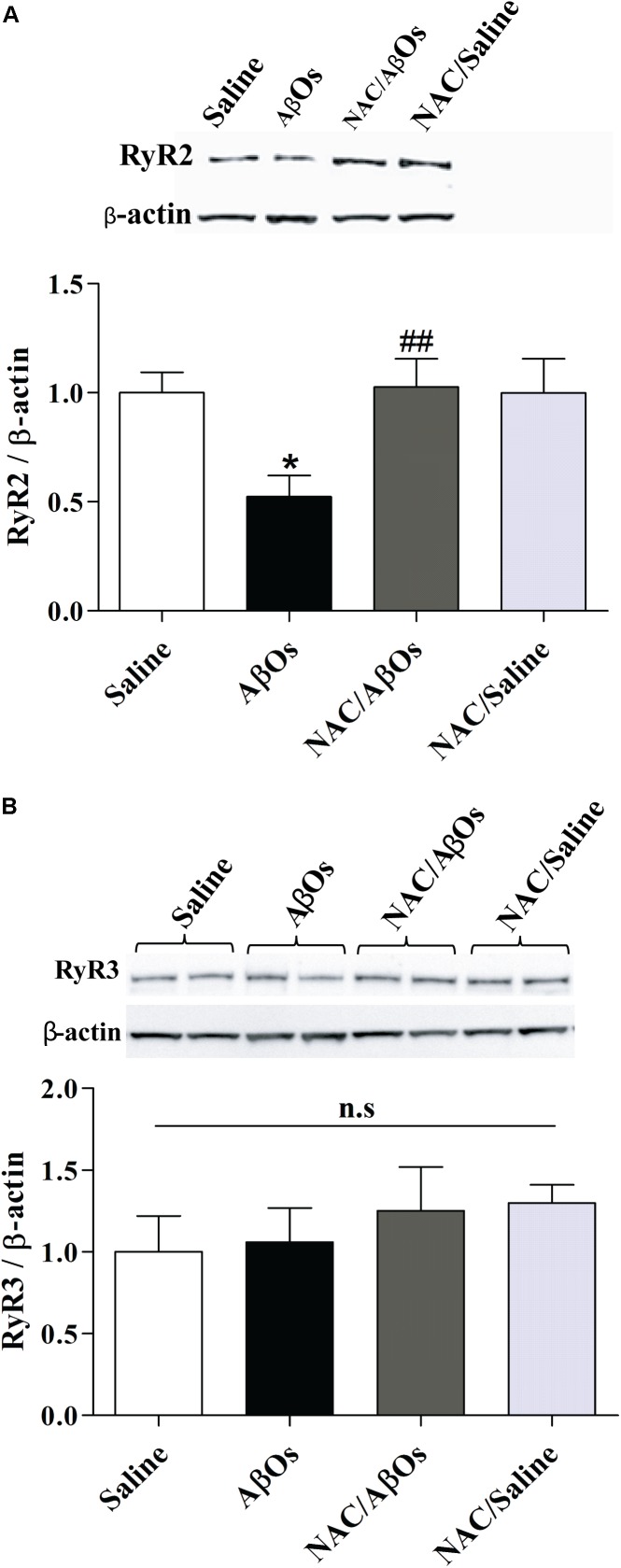
NAC feeding prevents the RyR2 downregulation induced by AβOs, without modifying RyR3 protein levels. The hippocampus of water deprived, spatial memory trained rats belonging to the following groups was removed and homogenized 1 h after the last training session. The four groups were: vehicle-fed rats injected with saline (control), vehicle-fed rats injected with AβOs (AβOs), NAC-fed rats injected with AβOs (NAC/AβOs), and NAC-fed rats injected with saline (NAC/Saline). **(A)**. Representative Western blot and relative quantification of RyR2 protein bands normalized to β-actin and expressed as fold of control. **(B)** Representative Western blot and relative quantification of RyR3 protein bands normalized to β-actin and expressed as fold of control. Values are expressed as mean ± SE (*n* = 3, for each group). Statistical analysis was determined by one-way ANOVA followed by Holm-Sidak *post hoc* test; ^∗^*p* < 0.05 respect to control; ^##^*p* < 0.01 respect to AβOs.

As reported earlier in primary hippocampal cultures exposed to AβOs (Paula-Lima et al., 2011), the relative RyR2 protein content was significantly lower in the whole hippocampus from trained rats injected with AβOs compared to trained rats injected with saline (Figure 9A). In contrast, the relative RyR2 protein contents of trained rats from the NAC/AβOs and the NAC/Saline groups were indistinguishable from the values exhibited by saline-injected rats exposed to the memory training protocol (Figure 9A). The relative RyR3 protein content was similar for the four groups (Figure 9B). These results show that AβOs downregulate RyR2 channels *in vivo* by engaging redox-sensitive pathways.

**FIGURE 10:**
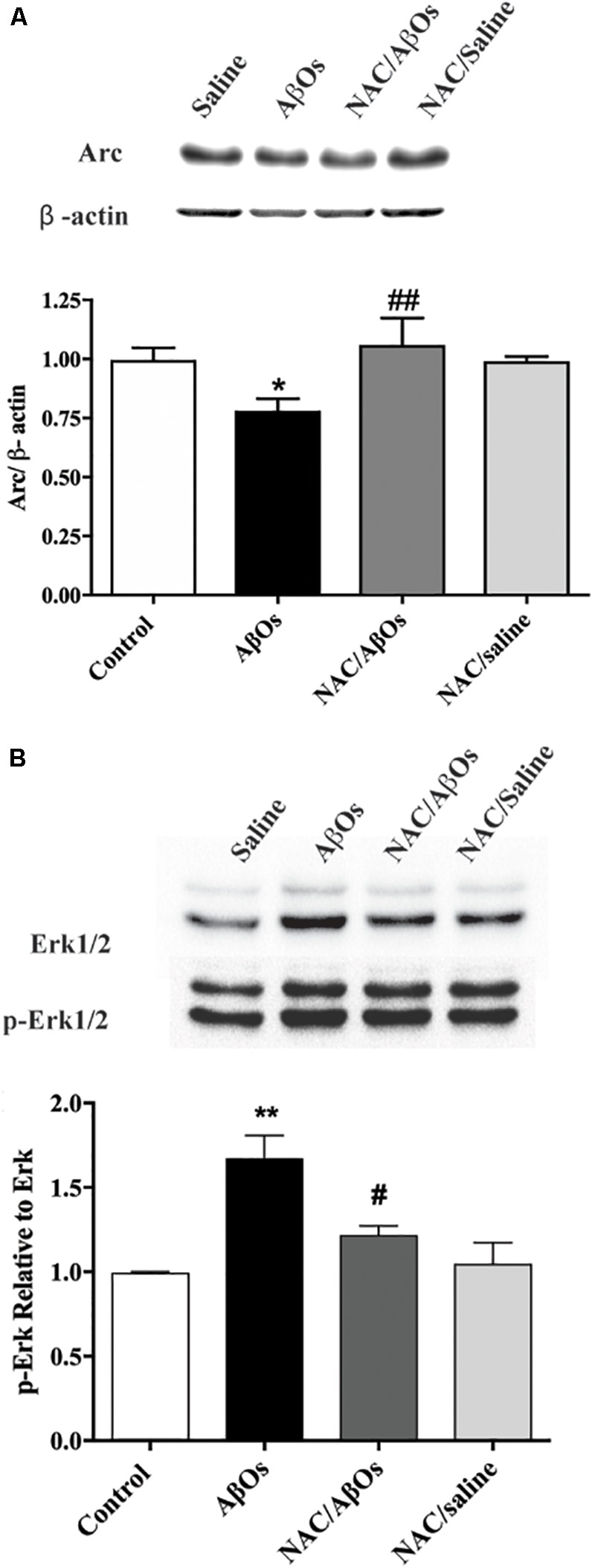
NAC feeding prevents the Arc downregulation and the enhanced ERK1/2 phosphorylation induced by AβOs injections. The hippocampus of water-deprived, spatial memory trained rats belonging to the following groups was removed and homogenized 1 h after the last training session. The four groups were: vehicle-fed rats injected with saline (control), vehicle-fed rats injected with AβOs (AβOs), NAC-fed rats injected with AβOs (NAC/AβOs), and NAC-fed rats injected with saline (NAC/Saline). **(A)** Representative Western blot and relative quantification of Arc protein bands normalized to β-actin and expressed as fold of control. **(B)** Representative Western blot and relative quantification of ERK1/2 phosphorylation relative to total ERK1/2 protein content. Values are expressed as mean ± SE (*n* = 3, for each group). Statistical analysis was determined by one-way ANOVA followed by Holm-Sidak *post hoc* test; ^∗^*p* < 0.05 respect to control; ^#^*p* < 0.05 and ^##^*p* < 0.01 respect to AβOs; ^∗∗^*p* < 0.01 respect to control.

Following AβOs injection, immunoblots revealed a significant decrease in Arc hippocampal protein content (Figure 10A) and a significant increase in ERK1/2 phosphorylation levels (Figure 10B); these changes did not occur in rats previously fed with NAC before AβOs injection. Rats fed with NAC and subsequently injected with saline displayed similar values as control (saline-injected) rats (Figure 10).

## Discussion

In the present study, we report that injections of AβOs into the rat CA3 hippocampal region impaired spatial learning and memory, decreased the hippocampal protein content of the immediate-early genes c-Fos and Arc, increased the fraction of single RyR channels responsive to Ca^2+^ activation, and decreased the total hippocampal RyR2 protein content without modifying RyR3 expression levels but increased RyR2 protein amounts in hippocampus-derived MAM fractions. We also report that oral administration of the general antioxidant NAC, a biological GSH precursor, preserved hippocampus redox potential and protected against the cognitive deficits, the GSH reduction and the RyR2 decrease induced by AβOs injections.

### Intrahippocampal AβOs-Injections Impair Spatial Memory in the Oasis Maze Task

Previous studies have described that i.c.v. injections of synaptotoxic AβOs cause deleterious effects on rat performance in different memory tasks and reproduce several AD pathological hallmarks – including gliosis, tau protein hyperphosphorylation and synapse loss (Townsend et al., 2006; Poling et al., 2008; Balducci et al., 2010; Dineley et al., 2010; Alberdi et al., 2013; Figueiredo et al., 2013; Lourenco et al., 2013; Forny-Germano et al., 2014; Brkic et al., 2015; Faucher et al., 2015; Ahmad et al., 2017). Moreover, direct stereotactic intra-hippocampal bilateral injections of AβOs in rodents induce microgliosis, synaptic degeneration, neuronal loss and hippocampal-dependent memory deficits (Zhong et al., 2009; Moon et al., 2011). Four sequential injections of AβOs into the dorsal hippocampus impair working memory and cause long lasting alterations of ERK1/2 signaling pathway in the medial prefrontal cortex and the septum, two brain areas tightly connected with the hippocampus and involved in working memory (Faucher et al., 2015).

Here, we show that three consecutive bilateral injections of AβOs into the CA3 region of rat hippocampus caused learning and memory deficits in the Oasis maze task, a dry version of the MWM spatial memory task (Clark et al., 2005). Altogether, these findings conclusively show that AβOs injections into the rodent hippocampus are sufficient by themselves to cause learning and memory impairments that correspond to the early stages of AD.

### Intrahippocampal AβOs-Injections Decrease c-Fos and Arc Protein Levels

The present results show that AβOs-injected rats displayed a drastic reduction of hippocampal c-Fos and Arc protein levels, two early gene products that have essential roles in hippocampal synaptic plasticity and memory processes (Vann et al., 2000; Guzowski et al., 2001; Ramirez-Amaya et al., 2005; Mamiya et al., 2009). In the hippocampus, the calcium-dependent rapid transcription of c-Fos and Arc occurs during hippocampal-dependent learning paradigms, including the MWM, novel environment exposure and contextual fear conditioning (Vann et al., 2000; Guzowski et al., 2001; Ramirez-Amaya et al., 2005; Mamiya et al., 2009; Minatohara et al., 2015). Accordingly, we propose that downregulation of these two proteins contributes to the inadequate performance of AβOs-injected rats in the Oasis maze spatial memory task. Of note, previous NAC feeding prevented the decrease of the Arc protein induced by AβOs injections. We interpret these results as a reflection of the enhanced oxidative environment induced by AβOs injection, which presumably by perturbing calcium signaling (Paula-Lima et al., 2011) downregulates Arc protein content. The protective effects of NAC feeding support this assumption.

Intra-hippocampal AβOs injections also promoted ERK1/2 phosphorylation; this increase was completely prevented by previous NAC feeding. These results add to previous finding showing that AβOs activate ERK1/2 in neuronal cell lines (Young et al., 2009) and rat primary neurons (Kirouac et al., 2017). Moreover, treatment of primary rat neurons with AβOs enhances ERK1/2 signaling, promoting GSK-3 activation and phosphorylation of the tau and APP proteins, resulting in amyloidogenic APP proteolysis and further Aβ generation (Kirouac et al., 2017). This chain of events generates a noxious feedforward mechanism, which by promoting tau hyperphosphorylation leads to neurodegeneration in AD (Kirouac et al., 2017). Based on our results, showing that NAC feeding prior to AβOs injection restores ERK1/2 phosphorylation to control levels, we strongly suggest that NAC prevents this harmful cycle.

### Intrahippocampal AβOs-Injections Decrease RyR2 Protein Levels

Several reports indicate that different memory processes require RyR-mediated Ca^2+^ release (for reviews, see Edwards and Rickard, 2006; Baker et al., 2013; Paula-Lima et al., 2014). In particular, RyR2 channels have a crucial role in hippocampal synaptic plasticity and spatial memory processes (Galeotti et al., 2008; More et al., 2018a). Altered RyR channel expression and function impact on the neuronal dysregulation displayed by rodent AD models (Stutzmann et al., 2006; Goussakov et al., 2010) and human AD patients (Kelliher, 1999). The increased RyR3 expression induced in primary cortical neurons by extracellular Aβ (Supnet et al., 2006) was proposed to play neuroprotective roles in the TgCRND8 mouse model of AD (Supnet et al., 2010). Treatment of cortical neurons with Aβ fibrils promotes ER Ca^2+^ release through RyR and IP_3_R channels, inducing ER stress, oxidative stress, and cell death (Ferreiro et al., 2008; Resende et al., 2008), while enhanced RyR recruitment contributes to Ca^2+^ disruptions in young, adult, and aged AD mice (Stutzmann et al., 2006), an effect of particular importance in dendritic spines (Goussakov et al., 2010). Neuronal RyR2 channels undergo *in vivo* post-translational remodeling (PKA-mediated phosphorylation, oxidation, and nitrosylation) in the brains of AD patients and two AD murine models (Lacampagne et al., 2017).

Previous results generated by our group have added additional information on the role of RyR channels in primary hippocampal neurons exposed to AβOs. In particular, we were the first to show (Paula-Lima et al., 2011) that AβOs decrease RyR2 but not RyR3 protein content in hippocampal neurons. Our findings revived the role of RyR2 channels in AD pathology reported in 1999 in the emblematic paper by Kelliher et al. (1999) since until our report the attention was focused on the RyR3 isoform. We have shown as well that RyR2-mediated Ca^2+^ release specifically partakes in the synaptic dysfunction caused by AβOs in primary hippocampal neurons (SanMartin et al., 2017).

The main contribution of the present work is the novel finding that AβOs injections intra-hippocampus caused a significant redox-dependent decrease in RyR2 protein content. These novel results support a pivotal role for the ROS-mediated decrease of hippocampal RyR2 channels as part of the mechanisms underlying the memory disruption produced by AβOs *in vivo*. The present findings, which are in accord with our previous *in vitro* results (Paula-Lima et al., 2011; Lobos et al., 2016; SanMartin et al., 2017) definitively constitute a substantial evidence that AβOs cause the RyR2 decrease reported in AD patients at early stages of the disease (Kelliher et al., 1999). Moreover, based on these combined findings it seems reasonable to propose that hippocampal-dependent memory loss in AD may be due – at least in part – to RyR2 downregulation, which would lead to the deficient generation of Ca^2+^ signals required for learning and memory processes.

It is important to highlight that this is the first report to describe *in vivo* the inhibitory effects of AβOs on RyR2 protein content in wild-type rats. All previous studies regarding RyR involvement were performed in transgenic AD models, which in addition to alterations in Aβ production also display changes in presenilins or Tau proteins that impede elucidating if the changes in RyR2 expression and function arise solely in response to AβOs accumulation. Moreover, the majority of AD sporadic cases involve abnormal brain accumulation of Aβ aggregates without alterations in APP, presenilins or Tau genes, whereas the inheritable familiar AD forms, which entail changes in these proteins, represent approximately 10% of the cases.

### AβOs Injection Promote the Emergence of Hippocampal Single RyR Channels That Display Enhanced Activation by Cytoplasmic [Ca^2+^]

Our previous studies (Marengo et al., 1996, 1998; Bull et al., 2003, 2008) indicate that oxidation or alkylation of critical SH residues enhances RyR single channel activation by cytoplasmic [Ca^2+^], as well as RyR-mediated CICR (Aracena-Parks et al., 2006). The present results, showing that RyR channels from AβOs-injected hippocampus displayed with higher frequency the moderate and high activity responses to Ca^2+^ are a strong indication that these RyR channels are more oxidized. In addition, we also show that NAC feeding partially prevented these changes in frequency distribution. The importance of our results resides in the fact that only channels responsive to activation by Ca^2+^, such as those that preferentially emerged after AβOs injections, are sufficiently activated by Ca^2+^ to sustain CICR at the Mg^2+^ and ATP concentrations of the cytoplasm (Bull et al., 2007). We propose that the enhanced hippocampal ROS production induced by AβOs (SanMartin et al., 2017; Munoz et al., 2018), which is prevented by NAC, increases RyR oxidation, a requisite condition to generate the cytoplasmic [Ca^2+^] increase caused by RyR-mediated CICR in response to AβOs-induced NMDA-mediated Ca^2+^ entry (Paula-Lima et al., 2011). Previous experiments, showing that RyR inhibition reduces the sustained [Ca^2+^] increase and the subsequent consequent deleterious effects in different experimental AD models, support the hypothesis that RyR channels mediate CICR in AD (Stutzmann et al., 2006; Paula-Lima et al., 2011; Sanmartin et al., 2012; SanMartin et al., 2017).

### NAC Counteracts the Spatial Memory Defects and the Decreased GSH/GSSG Ratios Induced by AβOs

Here, we show that NAC feeding for 21 days prevented the deleterious effects of AβOs injections on rat navigation in the Oasis maze spatial memory task. The deleterious effects of AβOs on memory have been associated with the fact that AβOs injected i.c.v. trigger pro-inflammatory pathways, including astrocyte and microglial activation (Moon et al., 2011; Alberdi et al., 2013; Lourenco et al., 2013; Forny-Germano et al., 2014; Brkic et al., 2015; Ahmad et al., 2017). We have recently shown that ROS released from AβOs-treated astrocytes elicit neuronal Ca^2+^ signals that promote inflammatory pathways (Munoz et al., 2018). Previous work has shown that the general antioxidant NAC, a GSH precursor that crosses the blood-brain-barrier (Katz et al., 2015), protects the hippocampus from oxidative stress, apoptosis, and Ca^2+^ entry (Naziroglu et al., 2014). We have reported that NAC inhibits the cytoplasmic Ca^2+^ signals (Paula-Lima et al., 2011), the mitochondrial fragmentation (Sanmartin et al., 2012) and the abnormal mitochondrial Ca^2+^ uptake and ROS increase (SanMartin et al., 2017) induced by AβOs in primary hippocampal neurons. Presumably, the effects of NAC on mitochondrial function are due to inhibition of excessive ROS-induced RyR-mediated CICR. Other studies indicate that NAC modulates inflammation and prevents cognitive and memory deficits in a rat model of traumatic brain injury (Haber et al., 2013). Oral supplementation with NAC also reverses the LTP abnormalities observed in aged animals (Robillard et al., 2011), and improves cognitive performance to various degrees in different neurodegeneration models, including AD (Huang et al., 2010; Hsiao et al., 2012; Shahidi et al., 2017). In transgenic APP/PS1 mice, NAC administration prevents passive avoidance memory impairment, suppresses brain protein oxidation and *S*-nitrosylation (Huang et al., 2010), decreases Aβ-40 and Aβ-42 hippocampal levels and restores contextual fear memory and hippocampal LTP (Hsiao et al., 2012). In addition, NAC treatment counteracts the impaired performance in a passive avoidance test and the electric alterations induced by intra-hippocampal Aβ injections in mice (Shahidi et al., 2017).

Cysteine availability is a limiting factor for GSH synthesis; it has been proposed that NAC treatment may increase cellular GSH levels and thereby counter oxidative stress, promote redox-regulated cell signaling, and improve immune responses (Hara et al., 2017). Here, we have confirmed previous reports indicating that NAC supplementation restores the decreased hippocampal GSH levels produced by i.c.v. injection of Aβ aggregates (Fu et al., 2006). The fact that NAC-fed animals injected with AβOs displayed similar GSH/GSSG ratios as controls is a strong indication that NAC reaches the hippocampus, where it preserves the redox homeostasis of this tissue despite the presence of toxic Aβ aggregates that significantly reduce the GSH/GSSG ratio in saline-fed rats injected with AβOs. In accord with the idea that GSH elevation could be a therapeutic strategy in AD treatment (Pocernich and Butterfield, 2012), we propose that the restoration of cellular oxidative tone induced by NAC is essential to counteract the oxidative stress induced by AβOs-injections, which is likely to promote non-physiological oxidation of redox-sensitive proteins, such as the RyR2 channels, which contribute to the memory deficits induced by AβOs.

### NAC Prevents the RyR2 Downregulation Caused by AβOs

Sub-lethal concentrations of AβOs decrease RyR2 protein content and prevent RyR-dependent BDNF- or caffeine-induced spine remodeling in primary hippocampal neurons (Paula-Lima et al., 2011). Here, we report that AβOs injections into the hippocampus down-regulated RyR2 but did not affect RyR3 levels, while oral NAC administration restored hippocampal RyR2 protein content without modifying RyR3 levels. These results complement our previous report showing that NAC protects primary hippocampal neurons from the decrease in *Ryr2* mRNA levels induced by AβOs (Lobos et al., 2016). We propose that NAC supplementation, which counteracts the RyR2 downregulation produced by AβOs injections, is an effective strategy to counteract the noxious effects of AβOs on memory processes. Interestingly, a recent clinical trial (ClinicalTrials.gov NCT01320527) performed in subjects who received a nutraceutical formulation containing NAC among other compounds showed that participants maintained their baseline cognitive performance over 12 months in contrast with the decline observed in placebo-receiving participants (Remington et al., 2016).

### RyR2 Is Present in MAM and Its Content Is Increased in the AβOs-Injected Hippocampus

The ER and mitochondria are in physical contact through MAM, which regulate in a concerted and bidirectional way cellular ROS and Ca^2+^ homeostasis and signaling (Seervi et al., 2013). Calcium transfer from the ER to the mitochondria relies on MAM-mediated physical contact between these two organelles. In the space between the ER and mitochondria, the intracellular Ca^2+^ concentration increases about 20-fold following Ca^2+^ release stimulation; the ER-mitochondria contact sites do not involve membrane fusion but entail a distance of approximately ∼20 nm between both organelles (Csordas et al., 2010). A proteomic study identified around 1,200 proteins in MAM fractions obtained from mouse brain (Poston et al., 2013). The presence of the inositol 1,4,5-trisphosphate (IP_3_) receptor (IP_3_R) – an ER-resident Ca^2+^ release channel – in MAM fractions from mouse brain has been previously reported (Hedskog et al., 2013). The MAM zone has a relevant role in the maintenance of ER redox conditions and various cellular processes, including apoptosis, regulation of cellular metabolism and mitochondrial function and dynamics (Raturi and Simmen, 2013).

In the context of AD, exposure of hippocampal neurons to the Aβ peptide increases the contact sites between the ER and mitochondria and leads to voltage-dependent anion channel (VDAC) and IP_3_R1 overexpression, resulting in augmented ER-to-mitochondria Ca^2+^ transfer (Hedskog et al., 2013). Up-regulation of MAM-associated proteins occurs in the human and mouse AD brain, in the APP-Swe/Lon mouse model and primary hippocampal cultures exposed to Aβ peptides (Hedskog et al., 2013). This up-regulation is likely to enhance mitochondrial Ca^2+^ uptake and ROS generation. Of note, no studies are available describing the presence of redox-sensitive RyR2 channels in hippocampal-derived MAM fractions. Interestingly, the proteins forming the γ-secretase complex (including presenilins 1 and 2) involved in APP cleavage are mostly concentrated in MAM fractions. Mutations in presenilins 1 and 2 and APP significantly increase ER-mitochondrial connectivity (Area-Gomez et al., 2012). Moreover, cells from patients with sporadic AD, in which presenilins 1 and 2 and APP structure are normal, display the same hallmarks of increased ER-mitochondria communication as do cells from familial AD patients (Schon and Area-Gomez, 2013). Therefore, a key role for the MAM region in mediating mitochondrial dysfunction in AD has been proposed (Area-Gomez et al., 2018). Alterations in MAM composition affect Ca^2+^ homeostasis (Csordas et al., 2010).

Here, we found that the hippocampal MAM fraction isolated from AβOs-injected rats is enriched in several known MAM-associated proteins, as previously described (Hedskog et al., 2013); in addition, we report for the first time the presence of RyR2 in hippocampal-derived MAM fractions. Surprisingly, although AβOs-injected rats displayed decreased hippocampal RyR2 protein content, the MAM fraction isolated from the hippocampus of these rats had higher RyR2 protein content relative to controls. We reported recently that RyR2-mediated Ca^2+^ release underlies the enhanced ER-mitochondrial Ca^2+^ transfer induced by AβOs in hippocampal neurons (SanMartin et al., 2017). Accordingly, we propose that RyR2 enrichment in the hippocampal MAM region will lead to increased ER-mitochondrial Ca^2+^ transfer and that the overall RyR2 downregulation displayed by AβOs-injected rats represents a compensatory change to limit mitochondrial Ca^2+^ overload. Downregulation of RyR2 channels, however, is likely to produce substantial memory impairments, since RyR2-mediated Ca^2+^ release is required for rodent hippocampal synaptic plasticity and spatial memory (More et al., 2018a). Further studies should address if these changes in cellular and MAM-associated RyR2 protein levels also occur in human AD patients.

## Conclusion

Here we show that daily oral NAC administration (200 mg/kg for 21 days) increased hippocampal total glutathione levels and GSH/GSSG ratios in AβOs or saline-injected rats compared to AβOs-injected rats not fed with NAC. Moreover, NAC feeding also prevented the spatial memory impairments and the RyR2 and Arc downregulation exhibited by rats injected with AβOs, evidencing the redox-dependence of these responses. These results strongly support previous proposals that NAC can be useful in the treatment of early AD patients.

## Author Contributions

JM is the Ph.D. student who led this work, executing most of the experiments. NG and PV were two Masters students who obtained the MAM and the results associated with these preparations. LM and GS were in charge of the western blots. JF and RB carried out the RyR single channel activity experiments. JV guided JM in behavioral experiments. CH participated in the design of the experiments and writing of the article with the corresponding author, AP-L, who in addition to writing the article and leading the work is responsible for the project that funded the work.

## Conflict of Interest Statement

The authors declare that the research was conducted in the absence of any commercial or financial relationships that could be construed as a potential conflict of interest.

## Abbreviations

AβOs: amyloid β peptide oligomers
ACSL4: acyl-CoA synthetase long-chain family member 4
AD: Alzheimer’s disease
APP: amyloid precursor protein
Arc: activity-regulated cytoskeleton-associated protein
BAPTA: 1,2-bis(o-aminophenoxy)ethane-N,N,N,N-tetraacetic acid
CA: *Cornu Ammonis* area
Ca^2+^: calcium ion
CICR: calcium-induced calcium release
CNX: calnexin
DAB: 3–3′diaminobenzidine hydrochloride
DAPI: 4,6-diamidino-2-phenylindole
DG: dentate gyrus
EDTA: ethylenediaminetetraacetic acid
EGTA: ethylene glycol-bis(β-aminoethyl ether)-N,N,N′,N′-tetraacetic acid
ER: endoplasmic reticulum
ERK: extracellular signal-regulated kinase
GSH: glutathione
GSSG: glutathione disulfide
HRP: horseradish peroxidase
i.c.v.: intracerebroventricular
Ig: immunoglobulin
IP_3_R1: inositol 1,4,5-trisphosphate receptor type-1
LTD: long-term depression
LTP: long-term potentiation
MAM: mitochondrial associated membranes
MOPS: 3-(N-morpholino)propanesulfonic acid
MWM: Morris water maze
NAC: N-acetylcysteine
NMDA: *N*-methyl-D-aspartate
PBS: phosphate buffered saline
PSD-95: postsynaptic density protein 95
PVDF: polyvinylidene difluoride
ROS: reactive oxygen species
RyR: ryanodine receptors
VDAC: voltage-dependent anion channel.
