# Supplementary figures and images for: N-Acetylcysteine Prevents the Spatial Memory Deficits and the Redox-Dependent RyR2 Decrease Displayed by an Alzheimer’s Disease Rat Model
Source: Front Aging Neurosci. 2018 Dec 6;10:399. doi: 10.3389/fnagi.2018.00399 (PMC6291746; doi:10.3389/fnagi.2018.00399)

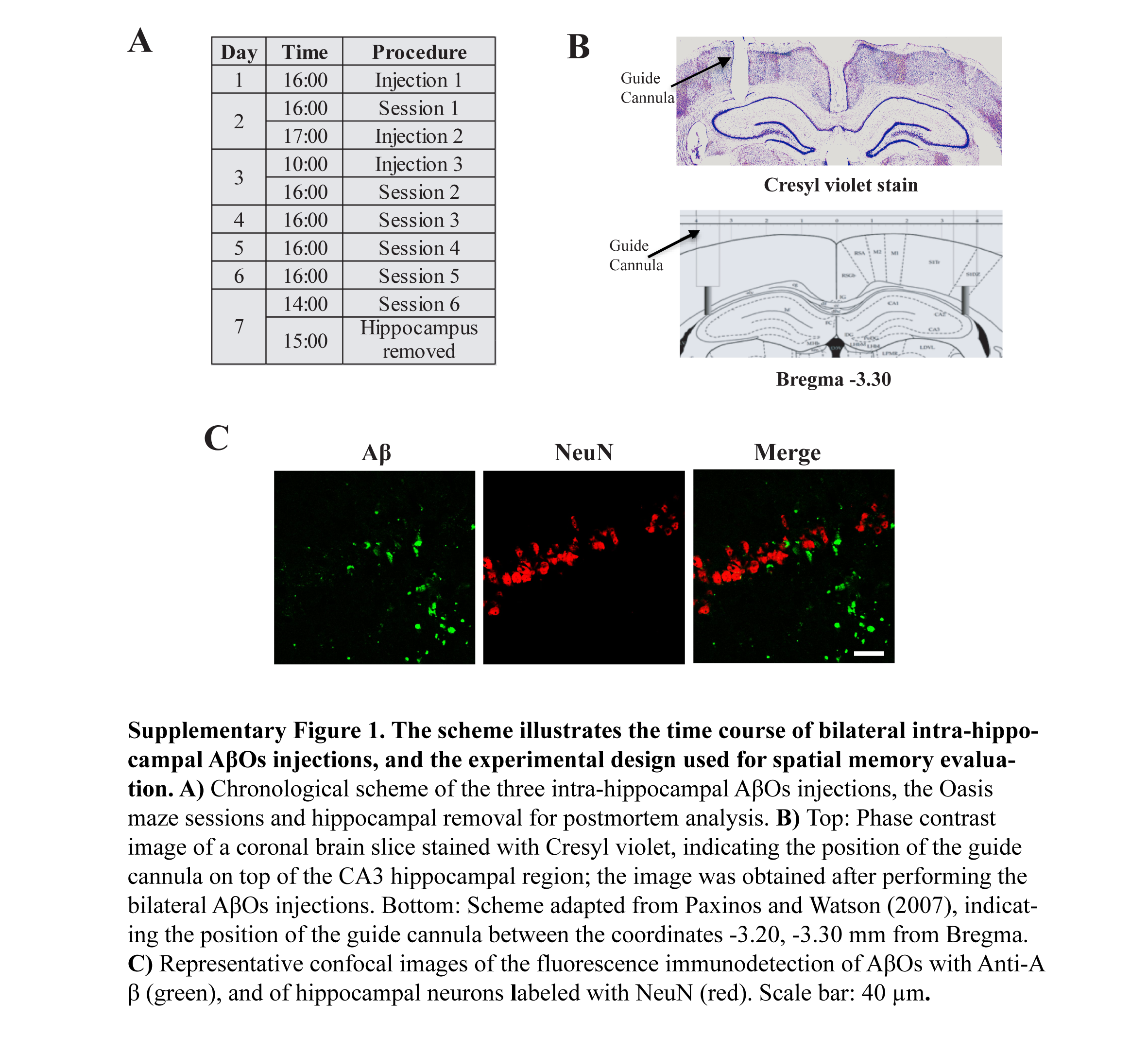

Supplement: Supplementary file 1 [file Image_1.tif]

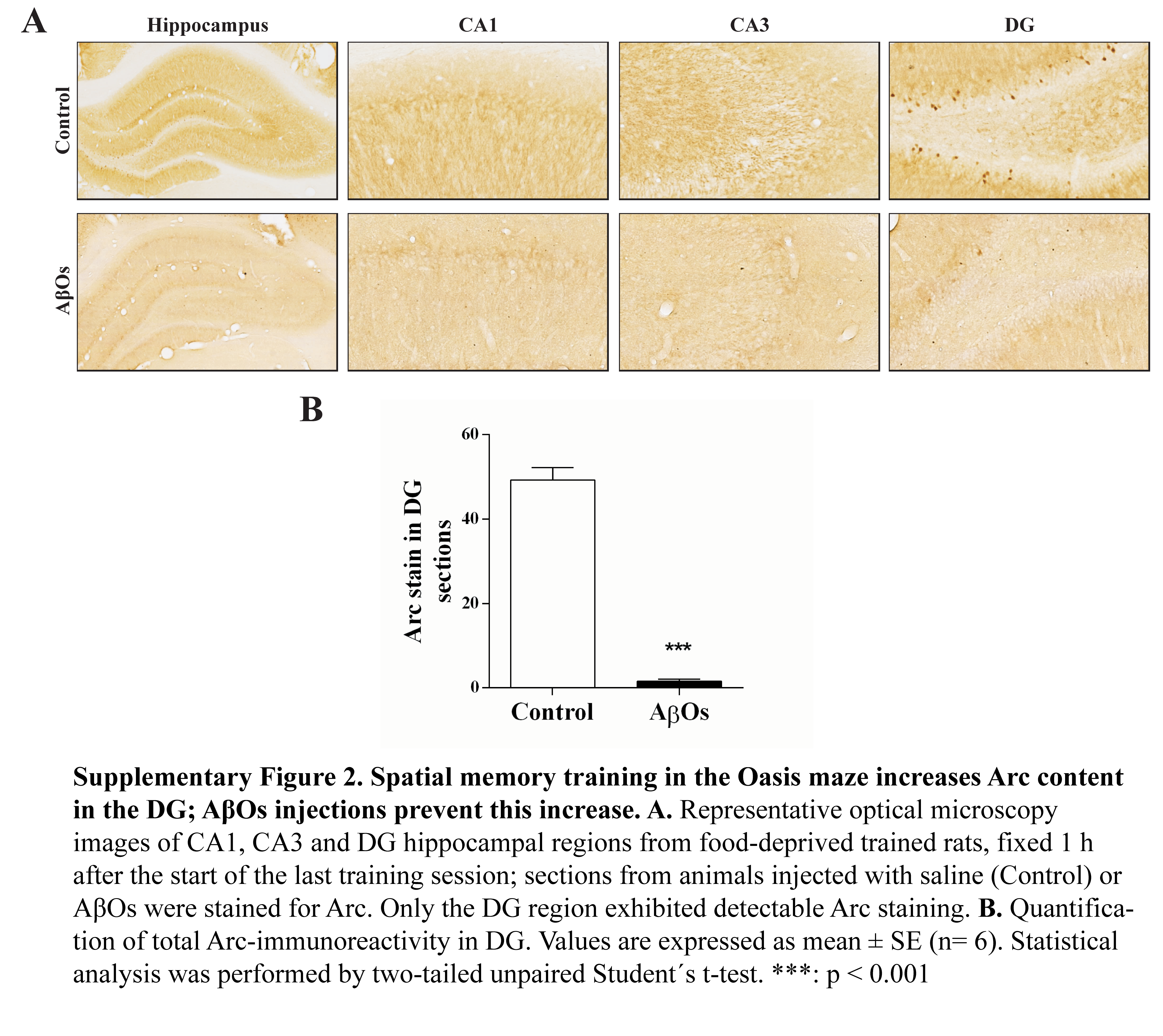

Supplement: Supplementary file 2 [file Image_2.TIF]

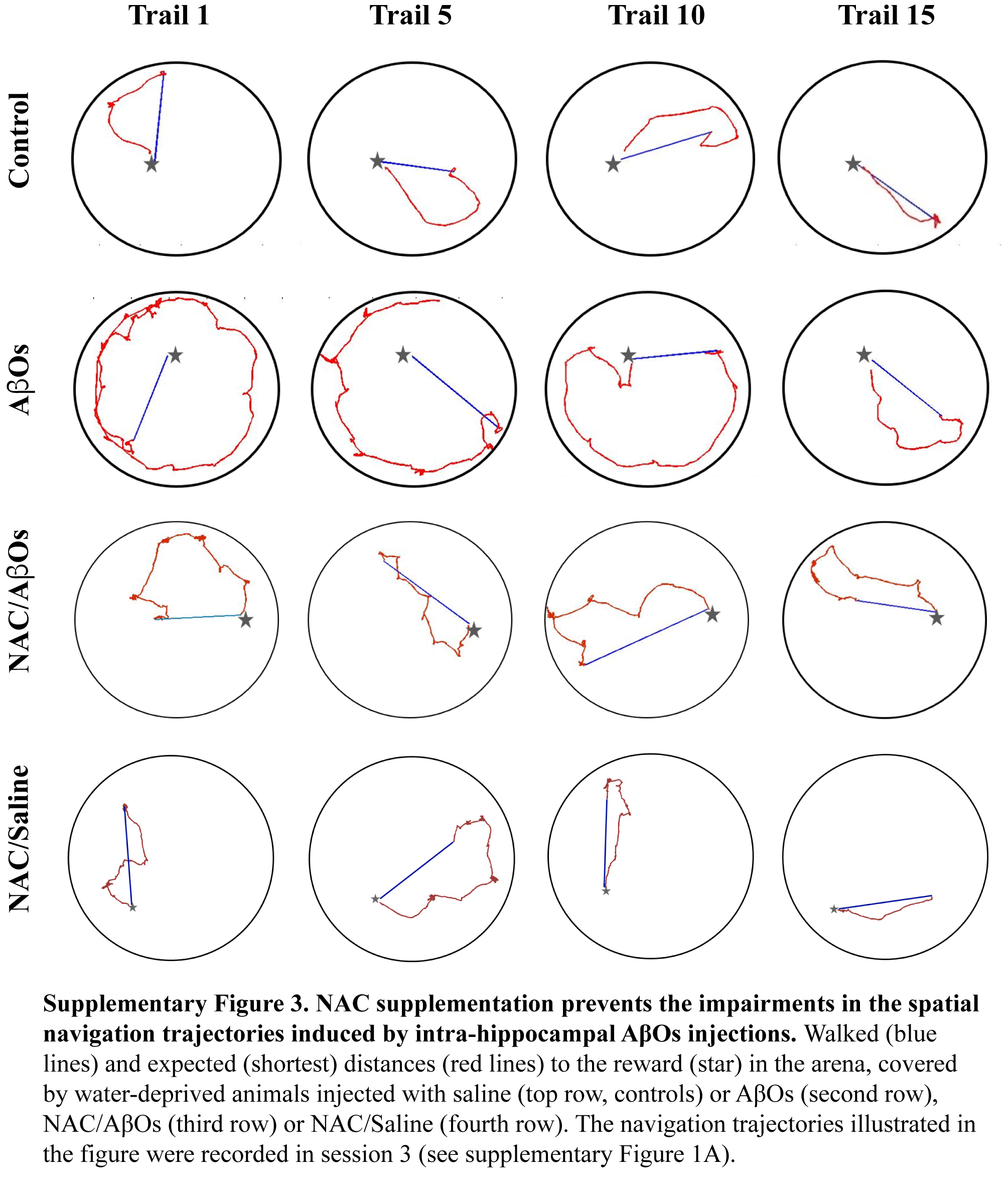

Supplement: Supplementary file 3 [file Image_3.TIF]
